# *Monodelphis domestica* Induced Pluripotent Stem Cells Reveal Metatherian Pluripotency Architecture

**DOI:** 10.3390/ijms232012623

**Published:** 2022-10-20

**Authors:** Satish Kumar, Erica M. De Leon, Jose Granados, Deanne J. Whitworth, John L. VandeBerg

**Affiliations:** 1 Department of Human Genetics and South Texas Diabetes and Obesity Institute, The University of Texas Rio Grande Valley School of Medicine, McAllen, TX 78504, USA; 2 Australian Institute for Bioengineering and Nanotechnology, University of Queensland, St Lucia, QLD 4072, Australia; 3 School of Veterinary Science, University of Queensland, Gatton, QLD 4343, Australia; 4 Department of Human Genetics and South Texas Diabetes and Obesity Institute, The University of Texas Rio Grande Valley School of Medicine, Brownsville, TX 78520, USA

**Keywords:** iPSC reprogramming, marsupial, pluripotency gene network

## Abstract

Marsupials have been a powerful comparative model to understand mammalian biology. However, because of the unique characteristics of their embryology, marsupial pluripotency architecture remains to be fully understood, and nobody has succeeded in developing embryonic stem cells (ESCs) from any marsupial species. We have developed an integration-free iPSC reprogramming method and established validated iPSCs from two inbred strains of a marsupial, *Monodelphis domestica.* The monoiPSCs showed a significant (6181 DE-genes) and highly uniform (*r*^2^ [95% CI] = 0.973 ± 0.007) resetting of the cellular transcriptome and were similar to eutherian ESCs and iPSCs in their overall transcriptomic profiles. However, monoiPSCs showed unique regulatory architecture of the core pluripotency transcription factors and were more like marsupial epiblasts. Our results suggest that *POU5F1* and the splice-variant-specific expression of *POU5F3* synergistically regulate the opossum pluripotency gene network. It is plausible that *POU5F1*, *POU5F3* splice variant XM_016427856.1, and *SOX2* form a self-regulatory network. *NANOG* expression, however, was specific to monoiPSCs and epiblasts. Furthermore, *POU5F1* was highly expressed in trophectoderm cells, whereas all other pluripotency transcription factors were significantly downregulated, suggesting that the regulatory architecture of core pluripotency genes of marsupials may be distinct from that of eutherians.

## 1. Introduction

Extant mammals include three major lineages: egg-laying monotremes (Prototheria), marsupials (Metatheria) and eutherian mammals (Eutheria). The most closely related of these are metatherian and eutherian mammals, sharing their most recent common ancestor ~160 million years ago (Mya), which is approximately half the time since the separation of mammals and birds (310–340 Mya) and about twice the time since the rapid radiation of eutherian species (66–90 Mya) [1,2,3]. Due to this unique evolutionary relationship with eutherians and birds, metatherians are frequently used as a comparative model to understand mammalian biology, as well as evolutionary events that gave rise to extant homeotherm species [4]. Research with marsupials has provided insights into the origins and evolutionary diversifications of mammalian-specific genetic and genomic structures, genomic imprinting, X-chromosome inactivation, immunobiology, neurobiology and carcinogenesis [4,5,6,7,8,9,10,11,12]. Since their evolutionary separation, metatherians and eutherians have developed some distinctive anatomic, physiologic, and genetic features, most notably in embryogenesis [13]. Unlike the eutherian blastocyst, the marsupial blastocyst forms initially as a bowl-shaped monolayer of cells lining the zona pellucida at the embryonic pole. Blastomeres from the embryonic pole then divide and expand in close contact with the zona pellucida, generating a unilaminar blastocyst that lacks a pluripotent inner cell mass. The blastomeres at the embryonic pole then act as precursors of the epiblast (EPI), the presumptive pluripotent cells, and of the hypoblast/primitive endoderm (PreE). Other blastomeres give rise to the trophectoderm (TE) [14,15]. Furthermore, the marsupial and monotreme genomes have retained *POU5F1* (also known as *OCT4*) and its paralogue *POU5F3* (also referred to as *POU2*); both are Class V POU transcription factors, and are important regulators of pluripotency, differentiation, and early development in vertebrates. *POU5F1* originated from a duplication event of ancestral *POU2* sometime in a common ancestor of cartilaginous and bony fishes during vertebrate evolution. Subsequently, either *POU5F1* or *POU5F3* was independently lost from the genomes of various lineages [16,17,18]. In mammals, *POU5F3* was lost only in eutherians [16]. Despite significant progress toward understanding morphological and transcriptional changes during early development in metatherians, including results from a recent single-cell RNA sequencing study [19], metatherian pluripotency architecture remains poorly understood. Due to the topographical differences between eutherian and metatherian blastocysts and many unknowns, nobody has succeeded in developing embryonic stem cells (ESCs) from any marsupial species. However, a successful iPSC reprogramming was reported from an Australian marsupial, the Tasmanian devil (*Sarcophilus harrisii*) [18].

To overcome these technological challenges to better understanding metatherian pluripotency and facilitating comparative biology and biomedically oriented research, we have developed integration-free induced pluripotent stem cells (iPSCs) from two fully inbred strains of the gray short-tailed opossum (*Monodelphis domestica*), the most widely used laboratory-bred research metatherian in the world. We performed deep RNA sequencing (RNASeq) of the validated *M. domestica* iPSCs (monoiPSCs) and conducted a comparative analysis with previously published transcriptomes of *M. domestica* embryonic and tissue-specific cell types. This is the first report of integration-free iPSC reprogramming from any metatherian species.

## 2. Results

### 2.1. monoiPSC Reprogramming and Validation

**Figure 1:**
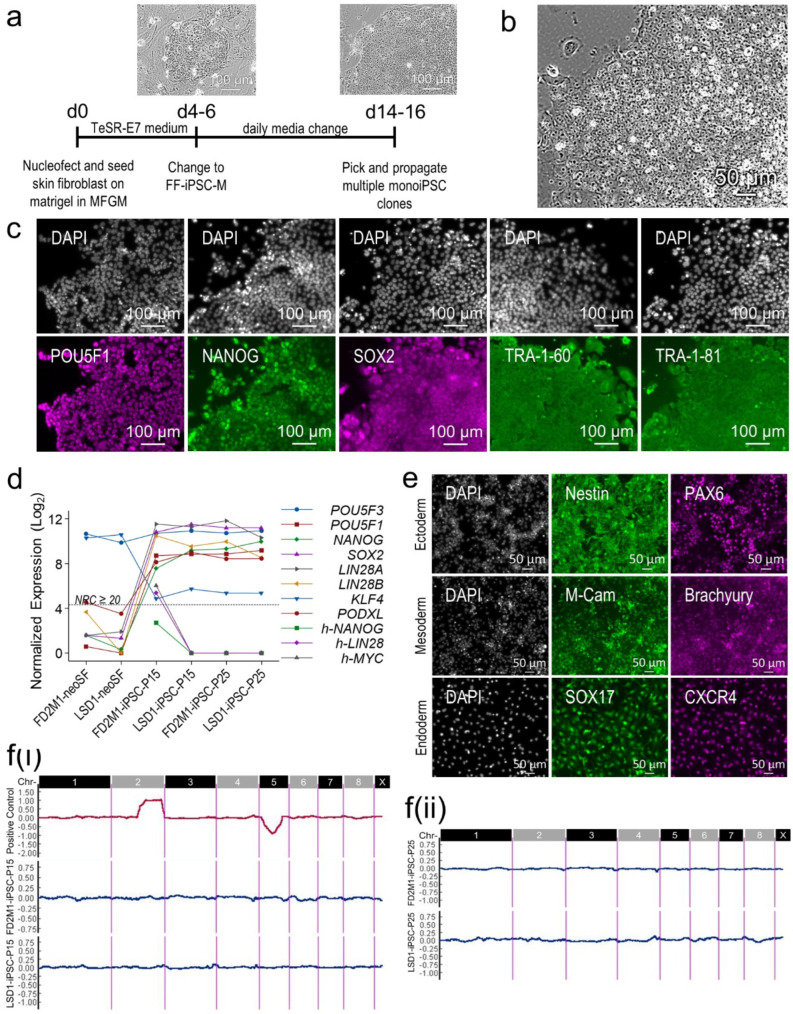
**monoiPSC reprogramming and validation.** (**a**) Schematic of monoiPSC reprogramming and stage-specific brightfield images of reprogramed cells. (**b**) Brightfield image of established monoiPSC colony (p25). (**c**) ICC expression analysis of the pluripotency markers in reprogrammed monoiPSCs. (**d**) Differential expression of endogenous pluripotency genes and human transgenes in neoSF and established monoiPSC clones. (**e**) Functional validation of established monoiPSCs by successful directed differentiation into the cells of all the three germ layers. (**f**) eKaryotyping moving average plots of regional biases in gene expression, validating a stable karyotype that was devoid of any structural aberrations in each established monoiPSC clone at (i) passage 15, and (ii) passage 25.

To develop monoiPSC lines, neonatal skin fibroblast (neoSF) cultures from 10-day-old female pups from two fully inbred strains, FD2M1 (*F* = 0.997) and LSD1 (*F =* 0.995) [20] were established. While in log growth phase, cells were nucleofected with each of the purified OriP/EBNA episomal plasmids, pCE-hOCT3/4, pCE-hUL, pCE-hSK, pCXLE-hMLN, pCE-mp53DD, and pCXB-EBNA1, encoding human reprogramming factors *OCT4*, *SOX2*, *NANOG*, *KLF4*, *LIN28* and *cMYC* [21,22]. We routinely reprogram human somatic cells into iPSCs without exogenous expression of *NANOG*; however, its exogenous expression was essential for successful reprogramming of monoiPSCs. The nucleofected cells were seeded on human embryonic stem cell (hESC)-qualified Matrigel Matrix (Corning)-coated six-well cell culture plates, allowed to recover in *Monodelphis* fibroblast growth medium (MFGM) for 18–24 h, and then transitioned into commercially available TeSR-E7 medium for reprogramming (Stem Cell Technologies Inc., Cambridge, MA, USA). From day 4 to day 6, when clusters of epithelial-like cells with large nuclei started to appear (Figure 1a), the reprogramming medium was replaced with feeder-free iPSC medium (FF-iPSC-M). It is important to note that extended culture in reprogramming medium results in apoptosis of the reprogrammed cells and may result in unsuccessful reprogramming. From day 14 to day 16, iPSC colonies with compact and flat colony morphology, and cells with high nucleus-to-cytoplasm ratio, were picked and expanded in feeder-free conditions in FF-iPSCM (Figure 1a,b). The established iPSC clones from both strains were analyzed by immunocytochemistry (ICC) and genome-wide RNA sequencing at passage 15 and passage 25, and showed robust expression and expected cellular localization of the pluripotency markers (Figure 1c,d).

The repertoire of endogenously expressed *M. domestica* pluripotency genes showed significant upregulation in reprogrammed iPSCs, with the exceptions of *POU5F3* and *KLF4*, which are both robustly expressed in *M. domestica* neoSFs (Figure 1d). *POU5F3* was robustly expressed in monoiPSCs as well; however, its expression did not change significantly during reprogramming. As reported previously [16] and discussed in the next section, expression of the *POU5F3* splice variant (XM_016427856.1), which encodes the full-length protein, seems to regulate pluripotency functions. This specific splice variant was significantly upregulated in monoiPSCs. Interestingly, *KLF2 and KLF4* were both stably expressed in all monoiPSC clones; however, their expression was significantly downregulated from the levels in neoSFs. *KLF* genes play an important role in eutherian pluripotency. In eutherians, *POU5F1* (*OCT4*) primarily induces *KLF2*, while the leukemia inhibitory factor (LIF)/STAT3 pathway selectively upregulates *KLF4*. Overexpression of either *KLF2* or *KLF4* reduces LIF dependency in eutherian embryonic stem cells (ESCs) and iPSCs [23]. The low expression of *KLF2* and *KLF4* plausibly explains the high LIF dependency observed in metatherian iPSCs. The iPSC cultures from the opossum and Tasmanian devil (*Sarcophilus harrisii*), the only two metatherian species from which reprogrammed iPSCs have been reported, are LIF-dependent [18]. Among the nucleofected human reprogramming transcription factors, nominal expression of *NANOG*, *LIN28* and *c-MYC* was detectable in one of the monoiPSC clones at passage 15, and become progressively silenced (likely due to the progressive depletion of the OriP/EBNA episomal plasmids) by passage 25 (Figure 1d). The monoiPSC clones established from both strains were successfully differentiated into cells of all the three germ layers, that is, Nestin- and PAX6-expressing neural progenitors (ectoderm), M-Cam- and Brachyury-expressing mesenchymal progenitors (mesoderm), and SOX17- and CXCR4-expressing definitive endodermal cells (endoderm), using directed differentiation in monolayer cultures (Figure 1e).

The monoiPSC clones of both strains at passage 15 and 25 were assessed for genomic integrity by e-karyotyping analysis of the genome-wide RNASeq data using CGH Explorer [24,25]. A sample with artificially generated chromosome 2 and 5 aneuploidies was used as positive control. Using the piecewise constant fit (PCF) algorithm implemented in CGH Explorer and a defined set of parameters detailed in the methods section, regional biases in gene expression were computed as a measure of structural aberrations. The results visualized using moving average plots showed that all tested monoiPSC clones had a stable karyotype that was devoid of any structural aberrations (Figure 1f).

### 2.2. Transcriptomic and Functional Architecture of monoiPSC

**Figure 2:**
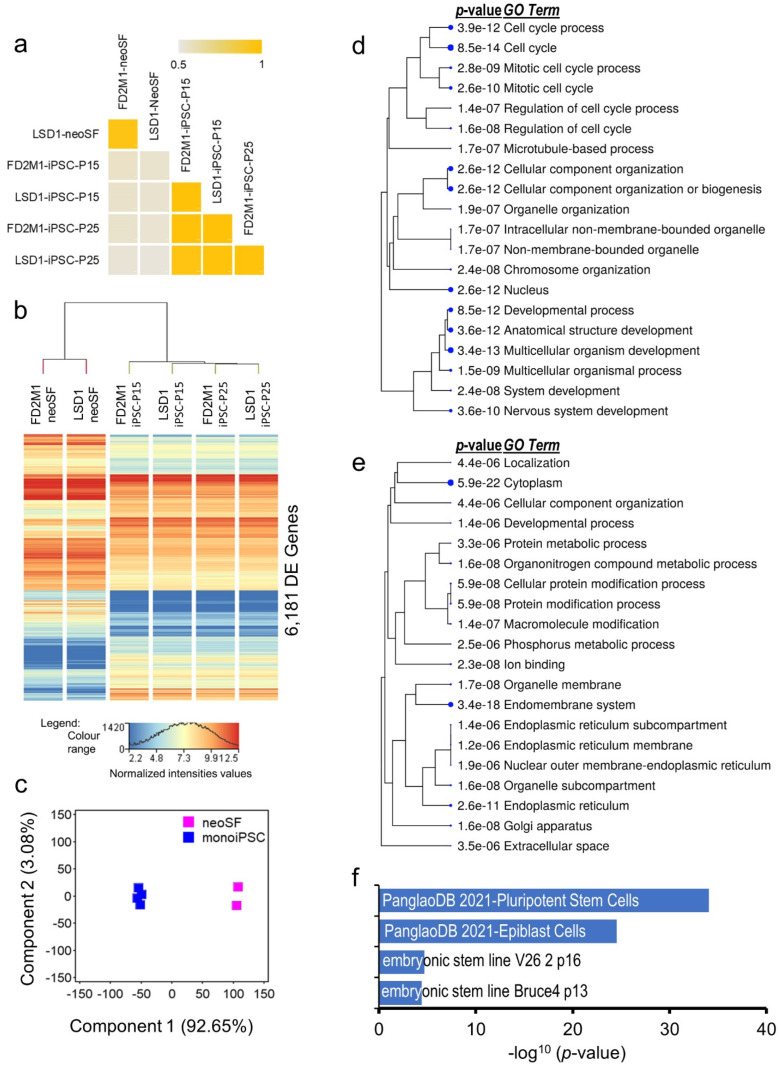
**monoiPSCs exhibit a large but highly uniform resetting of cellular transcriptome to pluripotency.** (**a**) Correlation (*r*^2^) plot of established neoSF and monoiPSC lines calculated based on their expressed transcriptome (13,599 genes having NRC ≤ 20 in neoSF and/or monoiPSCs). (**b**) Hierarchical clustering and heatmap of neoSF and their monoiPSC lines differentially expressed (DE) transcriptome (6181 genes). (**c**) Principal component analysis (PCA) of DE genes between neoSF and their monoiPSC lines. (**d**) Hierarchical clustering of the top 20 biological processes (*GO* terms) that were significantly enriched in monoiPSC significantly upregulated transcriptomes. (**e**) Hierarchical clustering of the top 20 biological processes (*GO* terms) that were significantly enriched in the neoSF transcriptome that was significantly downregulated in monoiPSCs. Both d and e gene ontology (GO) enrichment analyses were performed using ShinyGO v.0.75 web tool. GO terms are clustered based on shared genes; larger dot size indicates higher significance. (**f**) Bar plot showing highly significant enrichment of monoiPSC-upregulated transcriptome in PanglaoDB database [27] gene set for pluripotent stem cells and epiblast cells, and in mouse gene atlas of embryonic stem cell lines -V26 2p16 and -Bruce4 p13 9.

The *M. domestica* skin fibroblasts and iPSCs-expressed transcriptomes (genes with a normalized read count (NRC) ≥ 20 in all samples) comprised 12,284 and 12,256 genes, respectively, and showed an overlap of about 80%. The average correlation coefficient between four monoiPSC clones calculated based on their expressed transcriptomes (*r*^2^ at 95% CI = 0.973 ± 0.007) suggests highly concordant and reproducible transcriptomic architecture of the established monoiPSCs (Figure 2a). Genome-wide differential gene expression analysis of the neoSF and their reprogrammed monoiPSCs identified 6181 genes that were significantly (moderated *t* statistics FDR-corrected *p*-value ≤ 0.05 and fold change-absolute (FC-abs) ≥ 2.0) differentially expressed (DE) (Figure 2b; Supplementary Table S1). The DE transcriptome accounted for nearly 42% of the neoSF-expressed transcriptome and 41% of the monoiPSC-expressed transcriptome. About 93% of the observed variance in the DE genes was due to cellular transitions that occurred during the reprogramming of neoSFs into monoiPSCs (Figure 2c). These results suggest a large but highly concordant resetting of the cellular transcriptome, as is also observed in eutherian iPSC reprogramming [26]. The 2474 genes that were significantly upregulated in monoiPSCs showed significant enrichment in gene ontology (GO) processes, and cellular and molecular functions (Figure 2d) that were highly similar to eutherian ESC and iPSC functional profiles. Additionally, the monoiPSC-upregulated transcriptome showed highly significant enrichment in pluripotent stem cell (*p*-value = 8.4 × 10^−35^) and epiblast cell (*p*-value = 8.2 × 10^−19^) gene sets in the PanglaoDB database, which is a single-cell RNASeq database of mouse and human cells [27]; as well as in the mouse gene atlas of embryonic stem cell lines -V26 2p16 and -Bruce4 p13 9 (*p*-values = 2.1 × 10^−05^ and 4.0 × 10^−05^, respectively) (Figure 2f). The 3707 genes that were significantly downregulated during the reprogramming of neoSFs to monoiPSCs showed significant enrichment in skin-fibroblast-related cellular and molecular functions (Figure 2e). In PanglaoDB database [27] gene set enrichment analysis, the downregulated genes showed highly significant (the top) enrichment in the fibroblast gene set (*p*-value = 6.8 × 10^−34^), with an overlap of about 54% of genes.

### 2.3. POU5F1 and POU5F3 Synergistically Regulate Metatherian Pluripotency

**Figure 3:**
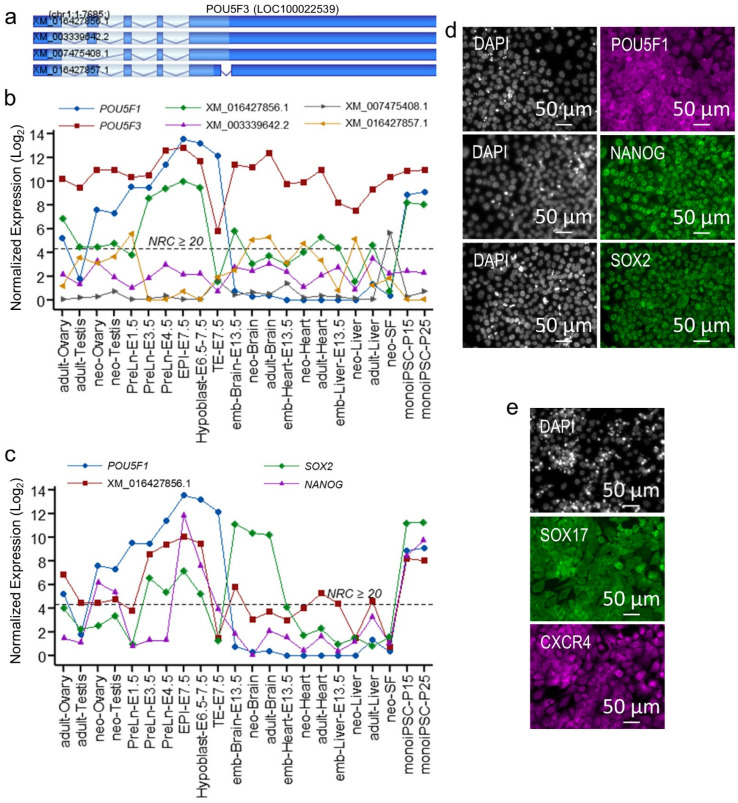
**POU5F1 and splice-variant-specific expression of POU5F3 synergistically regulate core pluripotency genes in monoiPSCs.** (**a**) Gene/exon structures of the *M. domestica* genome transcribed *POU5F3* splice variants; the XM_016427856.1 splice variant encodes full length transcript that is comprised of five exons; the XM_003339642.2 has exon 4 truncated and XM_007475408.1 lacks exon 4 completely; XM_16427857.1 has exon 1 truncated. (**b**) Line plot showing normalized expression of *POU5F1* and *POU5F3* splice variants in *M. domestica* embryonic cells, in brain, heart, liver, and gonadal developmental stages, and in neoSF and established monoiPSCs. (**c**) Line plot of core pluripotency transcription factors in *M. domestica* embryonic cells, in brain, heart, liver, and gonadal developmental stages, and in neoSF and established monoiPSCs. (**d**) ICC-based expression and localization of POU5F1, NANOG and SOX2 in a partially reprogrammed monoiPSC clone. (**e**) Successful differentiation of the partially reprogrammed monoiPSC clone into SOX17-expressing and CXCR4-expressing multipotent definitive endodermal cells.

As discussed in the introduction, *POU5F1* and *POU5F3* have both been conserved in marsupials; their continued presence over a long stretch of evolutionary history suggests that they likely have a role in marsupial pluripotency [16,18]. However, their expression patterns during embryonic development, as well as in adult tissues, remains intriguing [16,18,19]. To better understand the role *POU5F1* and *POU5F3* have in marsupial pluripotency, we examined the expression of *POU5F1*, and *POU5F3* and its splice variants in multiple *M. domestica* cell lineages during embryonic development and organ development, and in reprogrammed monoiPSCs, using data generated in this study and previously published data [19,28]. *POU5F3* was robustly expressed across all cell lineages examined, including embryonic cells and during brain, heart, liver, and gonad development, as well as in adult tissues, but not in TE where it was downregulated manyfold (Figure 3b). Notably, all *POU5F3* splice variants showed significant downregulation in TE, whereas splice-variant-specific expressions were observed in other cell/tissue types (Figure 3a,b). As mentioned above, splice variant XM_016427856.1, which encodes the full length *POU5F3* protein, was dominantly expressed in embryonic pre-lineage cells (PreLn) at embryonic day 3.5 (E3.5) and E4.5, in EPI (E7.5), in hypoblast (E6.5–7.5), and in reprogrammed iPSCs, and showed significant upregulation coinciding with embryonic genome activation around embryonic day 3.5 (E3.5) [19]. Interestingly, both *POU5F1* and XM_016427856.1 were nominally expressed in adult ovary; however, *POU5F1* expression significantly increased in PreLn-E1.5, suggesting the possibility that increased expression of *POU5F1* regulates the activation of the embryonic pluripotency network. The upregulated expression of XM_016427856.1 was restricted to the PreLn-E3.5-E4.5, EPI and hypoblast cells and to monoiPSCs, and was highly concordant with the expression of *SOX2* (Figure 3c). Unlike *POU* and *SOX2* transcription factors, high expression of *NANOG* was restricted to EPI (E7.5) and to reprogrammed monoiPSCs (*NANOG* was also expressed in hypoblast but showed significant downregulation compared to EPI), suggesting an EPI-like core pluripotency gene repertoire of monoiPSCs. Furthermore, continued expression of *POU5F1* in TE and significant downregulation of other core pluripotency transcription factors may suggest an alternate regulatory mechanism of the *POU5F1* function in these cells, probably the interaction of *POU5F1* and *CDX2* and/or exclusion of *POU5F1* from the nuclei [15,16]. During our reprogramming work, we identified some partially reprogrammed clones, which stably self-renewed in monoiPSC culture conditions and expressed POU5F1, SOX2 and NANOG proteins. However, unlike fully reprogrammed monoiPSCs, POU5F1 in these clones was localized to the cytoplasm (Figure 3d). These clones could differentiate into SOX17- and CXCR4-expressing endodermal cells with limited efficiency, but lacked the potential to differentiate into ectodermal and mesodermal lineages (Figure 3e), supporting an alternate regulatory mechanism of *POU5F1* function. Furthermore, these clones also established that *POU5F1* expression and its nuclear localization are essential for *M*. *domestica* pluripotency.

The high expression of *POU5F3* in other tissues (Figure 3b) showed a significantly different splice variant repertoire from that in pluripotent cells. While splice variant XM_016427856.1 was highly expressed in pluripotent cells and showed significant downregulation in other tissues, splice variant XM_016427857.1, which has a truncated exon 1, was upregulated and more or less compensated for the downregulated XM_016427856.1 expression in brain, heart, and liver tissues. In the neoSFs, however, splice variant XM_007475408.1, in which exon 4 and thus the part of DNA-binding POU domain is omitted, was dominantly expressed. What functions, counter-regulatory to each other or otherwise, these variants may play in these tissues, need further investigation, which can be facilitated by the generated monoiPSCs and their directed differentiation into different tissue types.

### 2.4. The monoiPSC Embryonic Lineage Marker Repertoire Is Akin to That of EPI

**Figure 4:**
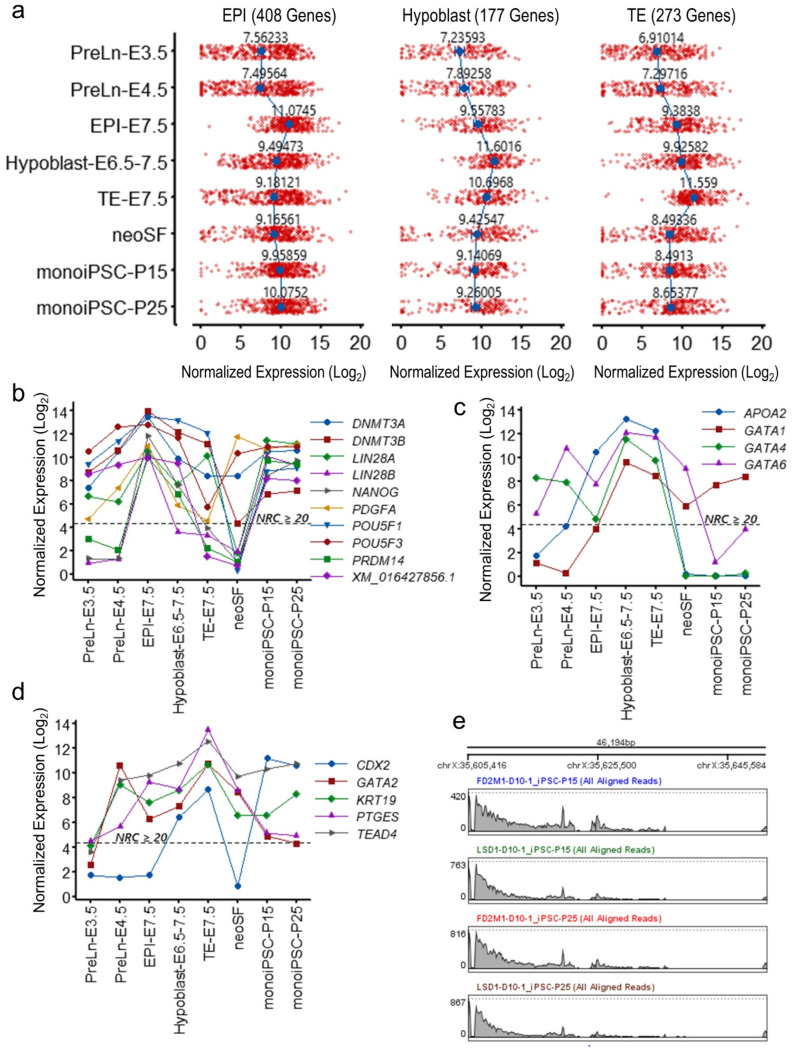
**monoiPSC embryonic lineage marker repertoire is akin to epiblasts.** (**a**) A comparative analysis of expression of *M. domestica* EPI, hypoblast/PreE and TE gene sets [19] in embryonic cells, neoSF and established monoiPSCs. (**b**) Expression of key EPI marker genes in embryonic cells, neoSF and monoiPSCs. (**c**) Expression of key hypoblast/PreE marker genes in embryonic cells, neoSF and monoiPSCs. (**d**) Expression of key TE marker genes in embryonic cells, neoSF and monoiPSCs. (**e**) Sequence alignment plots (raw read) showing robust transcription of the genomic region chrX: 35,605,415 to 35,651,609, in monoiPSCs. The genomic region transcribes *RSX* precursor RNA.

After having established that reprogrammed monoiPSCs have a core pluripotency gene repertoire similar to that of EPI, we examined the embryonic lineage-specific markers. Only single-cell RNASeq data was available for the EPI, hypoblast, and TE cell lineages. Because the total number of detected genes by single-cell RNASeq differs significantly from bulk RNASeq, transcriptome-wide comparisons were not feasible. Therefore, we focused this comparative analysis only on lineage-specific gene sets that were identified in a previous study [19], and that were annotated in the MonDom5 RefSeq genome assembly (Supplementary Table S2a–c). The expression profiles of these 408 EPI-, 177 hypoblast- and 273 TE-lineage-specific genes in pre-lineage (PreLn) -E3.5 and -E4.5, EPI-E7.5, hypoblast-E6.5-7.5, TE-E7.5, neoSF and monoiPS cells showed that reprogrammed monoiPSCs were akin to EPI (Figure 4a). To further evaluate these lineage-specific gene expression patterns in reprogrammed monoiPSCs, we focused on the key lineage-specific markers. The monoiPSCs showed an expression profile of key EPI markers (*POU5F1*, *POU5F3*, *NANOG*, *LIN28*, *PDGFA*, *PRDM14 and DNMT3A/B*) that was highly similar to that of EPI (Figure 4b). The expression of hypoblast/PreE marker genes (*GATA4*, *GATA6* and *APOA2*) was below the expression threshold of NRC ≥ 20 in monoiPSCs. The hypoblast lineage marker *GATA1* was expressed in monoiPSCs; however, its expression was significantly lower than in hypoblast cells (Figure 4c). The TE lineage markers *GATA2*, *PTGES and KRT19* were nominally expressed in monoiPSCs and were significantly downregulated as compared to TE cells at E7.5. *TEAD4* and *CDX2*, but were expressed in monoiPSCs (Figure 4d). In eutherians, *CDX2* acts downstream of *TEAD4* in TE development [29,30,31]. In opossum embryos, *POU5F1 (OCT4)*, *TEAD4* and *CDX2* proteins are co-expressed at nearly every stage; mutually exclusive localization of *POU5F1* and *CDX2* in the pluriblast and trophoblast, respectively, does not occur until well after opossum blastocyst expansion at ~ 256-cell stage, suggesting a complex interplay of *POU5F1* and *CDX2* expression and cellular localization in lineage specification [15]. Moreover, evidence suggests that *YAP1*-mediated activation of *CDX2* may play an important role in TE lineage specification [15]. Similar to embryonic pluriblast cells, the reprogrammed monoiPSCs show robust expression, as well as exclusively nuclear localization, of *POU5F1.*

Previous reports have shown that X-chromosome inactivation (XCI) occurs early in *M. domestica* embryogenesis and are maintained throughout embryogenesis in both non-EPI and EPI cells and into adulthood [19,32]. To assess the XCI status in reprogrammed monoiPSCs, we examined transcription of the genomic region within MonDom5 coordinates: chrX 35,605,415 to 35,651,609, which transcribe *RSX* precursor RNA. All established monoiPSC clones showed robust transcription in this region (Figure 4e), suggesting that the XCI is maintained in the reprogrammed monoiPSCs.

### 2.5. Transcriptional Architectures of monoiPSCs and Eutherian iPSCs Are Highly Similar

**Figure 5:**
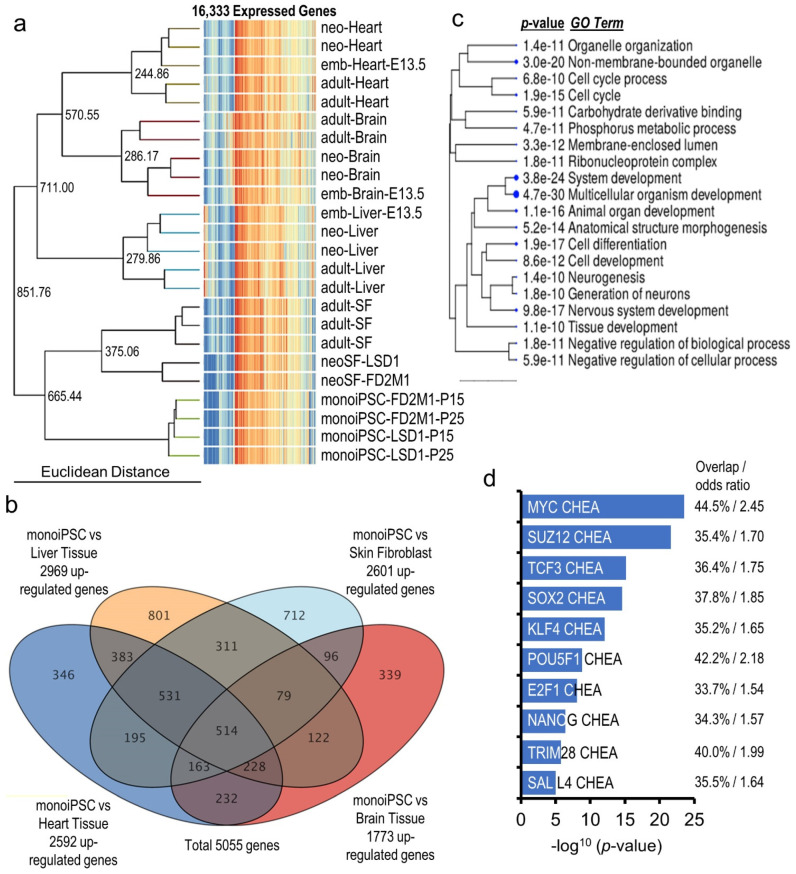
**Overall pluripotency transcriptional architecture is conserved between metatherian and eutherian mammals.** (**a**) Hierarchical clustering tree and heatmap of 16,333 genes that were expressed in monoiPSCs; in brain, heart, liver, and gonadal developmental stages; and in neonatal and adult skin fibroblasts shows a unique transcriptomic profile of monoiPSCs by comparison with terminally differentiated tissues. (**b**) Venn diagram of 5055 genes that were significantly upregulated in monoiPSCs as compared to one or more differentiated tissues/cell types. (**c**) Hierarchical clustering of top 20 biological processes (*GO* terms) that were significantly enriched in monoiPSC-upregulated transcriptome shows that monoiPSCs have a functional profile very similar to eutherian pluripotent cells. (**d**) The ChiP-X enrichment analysis (CHEA) [33] plot shows significant enrichment of the pluripotency transcription factor target genes in the monoiPSC upregulated transcriptome.

As discussed above, a significant resetting of the cellular transcriptome takes place during reprogramming of monoiPSCs. To better understand these changes and the transcriptomic architecture of marsupial pluripotency, we performed a transcriptome-wide comparison of monoiPSCs, and terminally differentiated skin fibroblasts, brain, heart, and liver tissue transcriptomes. Hierarchical clustering analysis of the monoiPSCs, skin fibroblasts, brain, heart, and liver expressed transcriptomes (16,333 genes having an NRC ≥ 20 in all samples of one or more cell type) shows that monoiPSCs retained a very limited transcriptional continuity from parental neoSF, and were significantly different from the terminally differentiated tissues (Euclidean distance (ED) of 665.44 with skin fibroblasts and 851.76 with all other tissues) (Figure 5a). To identify the monoiPSC gene set that plausibly constitutes the marsupial pluripotency architecture, we performed pair-wise differential gene expression analysis between monoiPSCs and the terminally differentiated tissues, including skin fibroblasts. A total of 5055 genes were significantly upregulated (moderated *t* statistics FDR-corrected *p*-value ≤ 0.05 and *FC* ≥ 2.0) in monoiPSCs as compared to one or more differentiated tissue/cell types: 2601 genes were significantly upregulated in monoiPSCs versus skin fibroblasts, 1773 genes versus brain tissue, 2592 genes versus heart tissue and 2969 genes versus liver tissue (Figure 5b; Supplementary Table S3a–d). These 5055 genes showed a highly significant enrichment (*p*-value = 1.2 × 10^−24^) and an overlap of 71.4% with the eutherian pluripotent stem cells gene set in the PanglaoDB database [27], suggesting a highly conserved pluripotency architecture between marsupials and eutherian mammals. *GO* term enrichment analysis also showed that the upregulated monoiPSC transcriptome has a functional profile similar to that of eutherian pluripotent stem cells (Figure 5c). The ChiP-X enrichment analysis (CHEA) [33] shows significant enrichment of the *MYC*, *POU5F1*, *SOX2*, *KLF4* and *NANOG* target genes in the identified 5055 gene set (Figure 5d). Overall, these results suggests that the pluripotency transcriptome of monoiPSCs is highly similar to that of eutherian iPSCs. 

## 3. Discussion

Here, we describe a transgene integration-free iPSC reprogramming method that we have developed for reprogramming *M. domestica* skin fibroblasts into iPSCs. We have established validated iPSC lines from two fully inbred strains and defined the transcriptomic landscape of the reprogrammed monoiPSCs to better understand the marsupial pluripotency architecture. A large but highly uniform resetting of the cellular transcriptome, similar to that which occurs in eutherian iPSC reprogramming, was observed [26]. The core pluripotency gene network and the functional profile of the generated monoiPSCs were highly similar to those of eutherian pluripotent cells (Figure 2). Within the embryonic lineages, monoiPSCs were similar to EPI as compared to PreLn, and lineage-committed hypoblast/PreE and TE cells (Figure 4a–d). The expression of pluripotency transcription factors *POU5F1*, *POU5F3* splice variant XM_016427856.1 and *SOX2* showed significant upregulation in PreLn cells at E3.5 and was highest in EPI, and also showed significant upregulation in monoiPSCs. Interestingly, *NANOG* expression, however, was more specific to EPI and monoiPSCs (Figure 3c). Furthermore, PreLn cells at E4.5 exhibited upregulated expression of lineage-specific markers (Figure 4b,c). These data suggest that the pluripotent state of *M. domestica* is highly similar to that of EPI. However, further comparative studies are necessary to identify the differences that may exist between monoiPSC and EPI cell states and to determine if a distinct naïve pluriblast population exists in marsupials [18,19]. Our data suggest a synergistic relationship between *POU5F1* and the *POU5F3* splice variant XM_016427856.1 in regulating the marsupial pluripotency gene network; a similar co-expression of *POU5F1*, *POU5F3*, *SOX2* and *NANOG* was reported in the iPSCs of the distantly related Tasmanian devil [18,34,35]. Plausibly *POU5F1*, the *POU5F3* splice variant XM_016427856.1 and *SOX2* form a self-regulatory pluripotency network similar to that of eutherians. However, *NANOG* expression that was specific to monoiPSCs and EPI, and high expression of *POU5F1* in TE, yet significant downregulation of all other pluripotency transcription factors suggest that the marsupial pluripotency network may have a distinct regulatory architecture than eutherians. The reprogramming methodology, monoiPSC lines, and data generated in this study will facilitate further investigations into some of these questions and will advance our understanding of mammalian pluripotency and its role in development and disease. 

*M. domestica* is the most widely used laboratory-bred research marsupial in the world and is the only marsupial that is available in large numbers for comparative biology and biomedical research [10]. The availability of monoiPSCs will significantly enhance the capability of exploiting the unique characteristics of this laboratory animal to better understand biological mechanisms involved in developmental and pathological processes [4,5,9,14,32,36,37], particularly since ESCs have not been established for *M. domestica* or any other marsupial species. Although the two monoiPSC lines that we developed from inbred strains are now available for research, the novel methods that we developed can be used to create integration-free iPSCs from any individual *M. domestica*. A potential practical application is the use of *M. domestica* in regenerative medicine research, such as stem cell therapy for the prevention or early-stage treatment of steatohepatitis, for which this species can serve as a unique model. Unlike mice, in which induction of non-alcoholic steatohepatitis (NASH) depends only on a homozygous mutant gene or on a diet containing extremely high levels of cholesterol (and usually with cholic acid), *M. domestica* resemble humans in that susceptibility to NASH is dependent on interaction of genotype and environment, specifically dietary cholesterol (without cholic acid) at a considerably lower level than required for the mouse model [36,38]. Another practical application, if our reprogramming methodology is successful with other marsupial species (as we expect it will be), is to apply it to research with endangered marsupial species. For example, iPSCs derived using a lentiviral vector from Tasmanian devils are being used in the development of a strategy to treat an infectious cancer which is threatening that species with extinction [18]. The use of integration-free iPSCs would reduce the risk of virus-induced insertional mutagenesis. It might also be practical to use integration-free iPSCs together with assisted reproductive technologies to increase the rate of captive propagation of those species via the generation of early-stage embryos from iPSCs. Already, blastocyst-like cells have been produced from human iPSCs [39]; however, significant technological hurdles are yet to be overcome. Overall, integration-free iPSCs that lack transgenes, some of which are oncogenic, are better suited for research and therapeutic applications.

In conclusion, the methodologies for creating integration-free iPSCs from marsupials, and the establishment of the first pluripotent stem cell lines from *M. domestica*, create a variety of new opportunities in fundamental research on mammalian pluripotency, regenerative medicine research involving stem cell therapies, and conservation research aimed at protecting endangered species.

## 4. Materials and Methods 

### 4.1. Data Reporting

No statistical methods were used to predetermine sample size. The experiments were not randomized, and investigators were not blinded to allocation during experiments and outcome assessment.

The study design specifically used female animals, keeping in view the future applications of the generated iPSC lines. For example, (1) iPSCs and iPSC-derived cells from females are not likely to be histo-incompatible when transplanted into male animals, whereas cells from males might be histo-incompatible when transplanted into female animals as a consequence of expression of a limited number of Y-linked genes; and (2) use of established iPSC lines in X-inactivation studies. Furthermore, our aim for this study was to develop an iPSC reprogramming methodology and established iPSC lines from this marsupial species to better understand marsupial pluripotency. Sex-specific differences do not significantly influence iPSC reprogramming or the overall pluripotency architecture, therefore were not considered in this study design.

### 4.2. Animal Maintenance and Sample Collection

The inbred strains from which the pups were harvested for this study were maintained under standard conditions [10] at The University of Texas Rio Grande Valley (UTRGV). All animal protocols used in this study were approved by the UTRGV’s Institutional Animal Care and Use Committee (IACUC). The breeders were maintained under IACUC-approved protocol AUP-19-31, and the pups were euthanized by decapitation under IACUC-approved protocol AUP-19-11. The bodies of the pups were then immersed and rinsed in 70% ethanol, and transferred in the ethanol to a biological safety cabinet. Skin on the back of the carcass was held away from the underlying muscle with a sterile forceps, and strips of skin were excised from beneath the forceps with a sterile scissors, and placed in MFGM.

### 4.3. Neonatal Skin Fibroblast Culture

Primary neoSF cultures from two fully inbred *M. domestica* strains, FD2M1 and LSD1 [20], were established using the methodology previously developed in our laboratory [40,41]. The established primary neoSF cultures were expanded for 3–4 passages in MFGM containing Iscove’s Modified Dulbecco’s Medium (IMDM), 16% fetal bovine serum (FBS), 0.5% insulin, transferrin, selenite supplement (ITS g supplement), 2 mM L-glutamine, 1X Antibiotic-Antimycotic solution (all from Gibco, ThermoFisher Scientific, Waltham, MA, USA) and 2 mM CaCl_2_ (Millipore Sigma, St. Louis, MO, USA).

### 4.4. monoiPSC Reprogramming

One million *M. domestica* neoSF cells in log growth phase were harvested and then nucleofected with 1 µg each of the purified OriP/EBNA episomal plasmids encoding human reprogramming factors *OCT4*, *SOX2*, *NANOG*, *KLF4*, *LIN28* and *cMYC* (all from Addgene, Watertown, MA, USA) [21,22], using Amaxa nucleofector technology on a 4D-Nucleofactor X Unit and the protocol (P2 primary cell nucleofector solution and DT130 program) we have optimized for *M. domestica* neoSF (Lonza, http://www.lonza.com/). The nucleofected neoSFs were seeded in human embryonic stem cell (hESC)-qualified Matrigel Matrix (Corning)-coated six-well cell culture plates in 2.0 mL MFGM per well. The cultures were incubated for 18–24 h undisturbed at 37 °C, 5% CO_2_ and atmospheric O_2_ and then transitioned into commercially available TeSR-E7 medium for reprogramming (Stem Cell Technologies Inc., Cambridge, MA, USA). When clusters of epithelial-like cells with large nuclei started to appear, usually around day 4–6 (Figure 1a), the reprogramming medium was replaced with FF-iPSC-M containing DMEM/F12, 1% N-2 supplement, 2% B-27 plus supplement, 2 mM GlutaMAX, 0.1 mM MEM NEAA, 10 mM HEPES buffer, 10 ng/mL human LIF, 50 ng/mL heat stable bFGF (all from Gibco, ThermoFisher Scientific, Waltham, MA, USA) and 0.25 µM Y27632 ROCK inhibitor, and 0.1 mM 2-mercaptoethanol (both from Millipore Sigma, St. Louis, MO, USA). Medium was changed daily. From day 14 to 16, iPSC colonies similar in morphology to human iPSCs (compact colonies and cells with high nucleus-to-cytoplasm ratio) were picked and expanded in feeder-free condition in FF-iPSC-M (Figure 1a,b). Established monoiPSC clones at passage 15 and 25 were characterized by ICC analysis of the pluripotent stem-cell markers and by genome-wide gene expression analysis (Figure 1c,d and Figure 2) using the methods detailed below.

### 4.5. monoiPSC Differentiation

To validate the differentiation potential of the monoiPSCs into the cells of all three germ layers, established monoiPSC clones were differentiated into neural stem cells, mesenchymal progenitor cells and definitive endodermal cells using the commercially available Gibco™ PSC Neural Induction Medium (Gibco, ThermoFisher Scientific, Waltham, MA, USA), STEMdiff™ Mesenchymal Progenitor Kit and STEMdiff™ Definitive Endoderm Kit (Stem Cell Technologies Inc., Cambridge, MA, USA) and manufacturers’ protocols. Minor modifications to each manufacturer-provided differentiation protocol were made and included optimization of initial seeding density of the monoiPSCs, confluency of the culture at the start of the differentiation and gradual transition of the iPSC culture from iPSC maintenance medium to differentiation medium. Successful differentiation of the monoiPSCs into neural stem cells, mesenchymal progenitor cells and definitive endodermal cells were assessed by cellular morphology analysis and ICC analysis of the lineage specific markers (Figure 1e). 

### 4.6. Immunocytochemistry Analysis

For ICC analysis of the pluripotency markers POU5F1, NANOG, SOX2, TRA-1-60 and TRA-1-81 in monoiPSCs, and lineage specific markers Nestin and PAX6 (ectoderm), M-Cam and Brachyury (mesoderm), and SOX17 and CXCR4 (endoderm) in the monoiPSC differentiated neural stem cells, mesenchymal progenitor cells, and definitive endodermal cells, cells were washed at room temperature with Dulbecco’s phosphate-buffered saline (DPBS) without CaCl_2_ and MgCl_2_ (Gibco, ThermoFisher Scientific, Waltham, MA, USA) and then fixed in 4% Paraformaldehyde (Millipore Sigma, St. Louis, MO, USA) for 15 min. After fixation, cells were washed three times with DPBS at room temperature, permeabilized for 12 min in permeabilization buffer containing 0.2% *v*/*v* Triton X-100 (Millipore Sigma, St. Louis, MO, USA), 1% *w*/*v* Bovine serum albumin (BSA; Millipore Sigma, St. Louis, MO, USA) in DPBS, and then blocked for an hour in blocking buffer containing 2% *w*/*v* BSA in DPBS and 5% *v*/*v* of serum from the species in which secondary antibodies were raised. Primary antibodies diluted to the manufacturer’s suggested concentration for immunofluorescence in blocking buffer were added to the cells, and the cells were incubated at 4 °C overnight. The primary antibodies were POU5F1/OCT4 (rabbit anti-human OCT4, PA5-27438, Invitrogen, ThermoFisher Scientific, Waltham, MA, USA), NANOG (mouse anti-human NANOG, MA1-017, Invitrogen, ThermoFisher Scientific, Waltham, MA, USA), SOX2 (rabbit anti-human SOX2, 3579, Cell Signaling Technology, Danvers, MA, USA), TRA-1-60 (mouse anti-human TRA-1-60, 4746, Cell Signaling Technology, Danvers, MA, USA), TRA-1-81 (mouse anti-human TRA-1-81, 4745, Cell Signaling Technology, Danvers, MA, USA), Nestin (mouse anti-human Nestin, 33475, Cell Signaling Technology, Danvers, MA, USA), PAX6 (rabbit anti-human PAX6, 60433, Cell Signaling Technology, Danvers, MA, USA), M-Cam (mouse anti-human M-Cam, 13475, Cell Signaling Technology, Danvers, MA, USA), Brachyury (rabbit anti-human Brachyury, 81694, Cell Signaling Technology, Danvers, MA, USA), SOX17 (mouse anti-human SOX17, MA5-24886, Invitrogen, ThermoFisher Scientific, Waltham, MA, USA) and CXCR4 (rabbit anti-human CXCR4, PA3-305, Invitrogen, ThermoFisher Scientific, Waltham, MA, USA). After overnight incubation in primary antibody solution, cells were washed three times for 5 min each with blocking buffer and then incubated for 1 h in donkey anti-mouse Alexa Fluor™ 488 and/or donkey anti-rabbit Alexa Fluor™ 594 (R37114 and R37119, respectively; Invitrogen, ThermoFisher Scientific, Waltham, MA, USA) secondary antibodies. Cells were washed three times for 5 min each with DPBS and counterstained with 5 μg/mL DAPI (4′,6-diamidino-2-phenylindole; Invitrogen) in DPBS for 2 to 3 min. Cells were rinsed with DPBS and then imaged immediately on a Carl Zeiss Axio Imager D2 research microscope. Appropriate negative controls were included in each ICC analysis. 

### 4.7. RNA Sequencing

Total RNA extracted from neoSF cells (~5 million cells), of the same passage that was used for reprogramming experiments, was sequenced in-house using Illumina’s TruSeq Stranded mRNA technology on an Illumina HiSeq 2500 instrument. Briefly, 1μg of high-quality neoSF total RNA per sample and the reagents supplied in Illumina TruSeq RNA sample preparation kit v2 (Illumina, Inc., San Diego, CA, USA) were used to prepare mRNA sequencing libraries. Poly-A tail containing mRNA molecules were first enriched from total RNA using oligo-dT-attached magnetic beads. The mRNA-enriched samples were then fragmented into ~200–600 base pair sizes using divalent cations and elevated temperature. The first-strand cDNA was synthesized from resulting cleaved RNA fragments and using reverse transcriptase and random primers, followed by second-strand cDNA synthesis using DNA polymerase-I and RNase H. The synthesized cDNA fragments were then end-repaired, and adaptor ligations were performed. The resulting cDNA libraries were purified, enriched by PCR and then deep-sequenced on an Illumina HiSeq 2500 platform. A total of 53 million, (FD2M1: 23,925,482; and LSD1: 29,066,534) 50 base-pair, single-end reads were obtained for neoSF samples. 

High-quality total RNA extracted from monoiPSCs (~5 million cells) established from FD2M1 and LSD1 *M. domestica* strains at passage 15 and 25, respectively, were sequenced using Novogene’s commercial sequencing service (Novogene Corporation Inc., Sacramento, CA, USA). A total of 190 million (FD2M1-P15: 46,427,592; FD2M1-P25: 43,071,188; LSD1-P15: 49,230,246; and LSD1-P25: 51,841,224) 150 base pairs, paired-end reads were obtained for monoiPSC samples. 

### 4.8. Additional Published RNA Sequencing Data

As mentioned in the results section, we used neoSF and monoiPSC RNASeq data generated in this study, as well as publicly available *M. domestica* single-cell RNASeq and bulk RNASeq data from previously published studies [19,28,42], for a comparative analysis to better understand marsupial pluripotency architecture. The published raw fastq files used in this study are described in Supplementary Table S4 and were downloaded from the NIH Sequence Read Archive (SRA). Briefly, the files included: (1) single-cell RNASeq files of 14 PreLn samples (four at E1.5, five at E3.5, and five at E4.5), ten EPI samples (at E7.5), ten hypoblast/PreE samples (at E6.5-7.5), and ten TE samples (at E7.5); and (2) bulk RNASeq files of three adult skin fibroblast samples, five each of brain, heart and liver samples (at E13.5 referred to as embryonic, post-birth day 10 and 14 referred to as neonatal, and post-birth day 90 and 180 referred to as adult), and four each of ovary and testis samples (two each neonatal and two each adult).

### 4.9. RNA Sequencing Analyses 

Raw fastq sequence files were generated and demultiplexed using Illumina bcl2fastq software (Illumina, Inc., San Diego, CA, USA) for the RNASeq data generated in-house. After pre-alignment quality controls (QCs) were conducted, the raw fastq files of neoSF, monoiPSC RNASeq data generated in this study, and other tissue RNASeq data downloaded from SRA (Supplementary Table S4), were aligned to *M. domestica* GCF_000002295.2 MonDom5 genome assembly and mapped to *M. domestica* RefSeq transcripts using StrandNGS software v4 (Strand Life Sciences Pvt. Ltd., Bangalore, India). Alignments were performed against transcriptome and genome together, and the discovery of novel slice sites was enabled. The aligned reads were filtered based on read quality metrics with the parameters set to quality threshold ≥ 20; Ns allowed in read ≤ 1; mapping quality threshold ≥ 40; and read length ≥ 20. Non-primary multiply mapped reads and reads failing the vendor’s QC were also removed. Transcript abundances were then computed by counting the number of reads that map to the genes and exons of interest using the algorithm implemented in StrandNGS software v4 and detailed in the StrandNSG reference manual available at the StrandNGS website (https://www.strand-ngs.com). The expression values (read counts) were log transformed, and “DESeq” normalization [43] was performed across all samples before any comparative/differential gene expression analysis. Known mRNAs/genes having NRC ≥ 20 in all samples of a cell/tissue type as specified in the results were considered expressed and selected for differential gene expression analysis.

### 4.10. eKaryotyping

Genomic integrity of the established monoiPSC lines was assessed by ekaryotyping analysis of the genome-wide RNASeq data using CGH Explorer and the method described in Weissbein et al., 2016, with minor modifications [24,25]. Briefly, the 12,256 genes that were expressed (NRC ≥ 20) in monoiPSCs were considered for eKaryotyping analysis. First, the median expression of each gene across all monoiPSC clones was subtracted from the expression value in each sample to obtain relative expression values. The median values then served as the baseline for examining expression bias. To reduce expression noise further, the sum of squares of the relative expression values was calculated for each gene, and the 10% most variable genes were removed from the analysis data set. The regional biases in gene expression were then detected using the piecewise constant fit (PCF) algorithm implemented in CGH Explorer and a defined set of parameters: least allowed deviation = 0.25; least allowed aberration size = 50; Winsorize at quantile = 0.001; penalty = 12; and threshold = 0.01. The results were than visualized by moving average plots with windows of 200 genes in the moving average fit tool implemented in the CGH Explorer. A sample with artificially generated chromosome 2 and 5 aneuploidies was used as positive control. 

### 4.11. Differential Gene Expression Analyses

To identify genes/mRNAs that were DE between monoiPSCs and cell/tissue of interest, moderated *t* statistics and expression fold change analyses were performed. The genes/mRNAs having moderated *t* statistics FDR-corrected *p*-value ≤ 0.05 and FC-abs ≥ 2.0 between the pair of sample types were considered significantly differentially expressed.

### 4.12. Functional Annotations and Enrichment Analyses

Functional annotation and enrichment analyses of a gene list of interest, as detailed in the results, were performed using “Enrichr” and “ShinyGO v0.75”; both of these web tools are publicly available for gene list enrichment analyses [44,45,46]. At the time of enrichment, analyses presented in this study “Enrichr” included 382,985 functional terms and 193 gene set libraries constructed from many sources, such as published studies and major biological and biomedical online databases. The CHEA library used in this study is a set of functional terms representing transcription factors profiled by ChIP-seq in mammalian cells and is only available through Enrichr. Enrichr implements a number of enrichment scores described in Chen et al., [44]; we ranked our Enrichr-significant results based on computed Fisher exact test *p*-values. ShinyGO allows in-depth analysis of gene lists, with graphical visualization of enrichment, pathway, gene characteristics, and protein interactions. At the time of the enrichment analyses presented here, “ShinyGO v0.75” included GO terms and pathways for over 400 animal and plant species, based on annotation from Ensembl, Ensembl plants, and Ensembl Metazoa data bases. An additional 5090 species genomes were also annotated based on STRINGdb v. 11.5. In ShinyGO enrichment analyses; we used the “best matching species” option to query our list of genes of interest, and Chi-square test FDR corrected *p*-values ≤ 0.05 were used for statistical significance. Significant enrichment into the top 20 functional terms were visualized by constructing hierarchical clustering tree(s).

## Data Availability

The mRNA sequence data generated from two *M. domestica* neoSF samples and four monoiPSC clones were submitted to the National Center for Biotechnology Information Gene Expression Omnibus (GEO) archive and are accessible through the GEO Series accession number GSE206122. All other relevant data are available from the corresponding author on request.

## Supplementary Materials

The following supporting information can be downloaded at: https://www.mdpi.com/article/10.3390/ijms232012623/s1.

## References

[1] Halliday T.J., Upchurch P., Goswami A. (2016). Eutherians experienced elevated evolutionary rates in the immediate aftermath of the Cretaceous-Palaeogene mass extinction. Proc. Biol. Sci..

[2] Hedges S.B., Kumar S. (2004). Precision of molecular time estimates. Trends Genet..

[3] Luo Z.X., Yuan C.X., Meng Q.J., Ji Q. (2011). A Jurassic eutherian mammal and divergence of marsupials and placentals. Nature.

[4] Samollow P.B. (2008). The opossum genome: Insights and opportunities from an alternative mammal. Genome Res..

[5] Das R., Anderson N., Koran M.I., Weidman J.R., Mikkelsen T.S., Kamal M., Murphy S.K., Linblad-Toh K., Greally J.M., Jirtle R.L. (2012). Convergent and divergent evolution of genomic imprinting in the marsupial Monodelphis domestica. BMC Genom..

[6] Graves J.A., Renfree M.B. (2013). Marsupials in the age of genomics. Annu. Rev. Genom. Hum. Genet..

[7] Mahadevaiah S.K., Royo H., VandeBerg J.L., McCarrey J.R., Mackay S., Turner J.M. (2009). Key features of the X inactivation process are conserved between marsupials and eutherians. Curr. Biol. CB.

[8] Murchison E.P., Schulz-Trieglaff O.B., Ning Z., Alexandrov L.B., Bauer M.J., Fu B., Hims M., Ding Z., Ivakhno S., Stewart C. (2012). Genome sequencing and analysis of the Tasmanian devil and its transmissible cancer. Cell.

[9] Saunders N.R., Noor N.M., Dziegielewska K.M., Wheaton B.J., Liddelow S.A., Steer D.L., Ek C.J., Habgood M.D., Wakefield M.J., Lindsay H. (2014). Age-dependent transcriptome and proteome following transection of neonatal spinal cord of Monodelphis domestica (South American grey short-tailed opossum). PLoS ONE.

[10] VandeBerg J., Williams-Blangero S., Hubrecht R., Kirkwood J. (2010). The Laboratory Opossum. The UFAW Handbook on the Care and Management of Laboratory and Other Research Animals.

[11] VandeBerg J.L., Robinson E.S. (1997). The laboratory opossum (*Monodelphis domestica*) in laboratory research. ILAR J..

[12] VandeBerg J.L., Williams-Blangero S., Hubbard G.B., Ley R.D., Robinson E.S. (1994). Genetic analysis of ultraviolet radiation-induced skin hyperplasia and neoplasia in a laboratory marsupial model (Monodelphis domestica). Arch. Dermatol. Res..

[13] Frankenberg S.R., de Barros F.R., Rossant J., Renfree M.B. (2016). The mammalian blastocyst. Wiley Interdiscip. Rev. Dev. Biol..

[14] Frankenberg S., Shaw G., Freyer C., Pask A.J., Renfree M.B. (2013). Early cell lineage specification in a marsupial: A case for diverse mechanisms among mammals. Development.

[15] Morrison J.T., Bantilan N.S., Wang V.N., Nellett K.M., Cruz Y.P. (2013). Expression patterns of Oct4, Cdx2, Tead4, and Yap1 proteins during blastocyst formation in embryos of the marsupial, Monodelphis domestica Wagner. Evol. Dev..

[16] Frankenberg S., Pask A., Renfree M.B. (2010). The evolution of class V POU domain transcription factors in vertebrates and their characterisation in a marsupial. Dev. Biol..

[17] Frankenberg S.R., Frank D., Harland R., Johnson A.D., Nichols J., Niwa H., Scholer H.R., Tanaka E., Wylie C., Brickman J.M. (2014). The POU-er of gene nomenclature. Development.

[18] Weeratunga P., Shahsavari A., Ovchinnikov D.A., Wolvetang E.J., Whitworth D.J. (2018). Induced pluripotent stem cells from a marsupial, the Tasmanian Devil (*Sarcophilus harrisii*): Insight into the evolution of mammalian pluripotency. Stem Cells Dev..

[19] Mahadevaiah S.K., Sangrithi M.N., Hirota T., Turner J.M.A. (2020). A single-cell transcriptome atlas of marsupial embryogenesis and X inactivation. Nature.

[20] Xiong X., Samollow P.B., Cao W., Metz R., Zhang C., Leandro A.C., VandeBerg J.L., Wang X. (2022). Genetic and genomic architecture in eight strains of the laboratory opossum Monodelphis domestica. G3.

[21] Okita K., Matsumura Y., Sato Y., Okada A., Morizane A., Okamoto S., Hong H., Nakagawa M., Tanabe K., Tezuka K. (2011). A more efficient method to generate integration-free human iPS cells. Nat. Methods.

[22] Okita K., Yamakawa T., Matsumura Y., Sato Y., Amano N., Watanabe A., Goshima N., Yamanaka S. (2013). An efficient nonviral method to generate integration-free human-induced pluripotent stem cells from cord blood and peripheral blood cells. Stem Cells.

[23] Hall J., Guo G., Wray J., Eyres I., Nichols J., Grotewold L., Morfopoulou S., Humphreys P., Mansfield W., Walker R. (2009). Oct4 and LIF/Stat3 additively induce Kruppel factors to sustain embryonic stem cell self-renewal. Cell Stem Cell.

[24] Lingjaerde O.C., Baumbusch L.O., Liestol K., Glad I.K., Borresen-Dale A.L. (2005). CGH-Explorer: A program for analysis of array-CGH data. Bioinformatics.

[25] Weissbein U., Schachter M., Egli D., Benvenisty N. (2016). Analysis of chromosomal aberrations and recombination by allelic bias in RNA-Seq. Nat. Commun..

[26] Kumar S., Curran J.E., Glahn D.C., Blangero J. (2016). Utility of lymphoblastoid cell lines for induced pluripotent stem cell generation. Stem. Cells Int..

[27] Franzen O., Gan L.M., Bjorkegren J.L.M. (2019). PanglaoDB: A web server for exploration of mouse and human single-cell RNA sequencing data. Database.

[28] Cardoso-Moreira M., Halbert J., Valloton D., Velten B., Chen C., Shao Y., Liechti A., Ascencao K., Rummel C., Ovchinnikova S. (2019). Gene expression across mammalian organ development. Nature.

[29] Strumpf D., Mao C.A., Yamanaka Y., Ralston A., Chawengsaksophak K., Beck F., Rossant J. (2005). Cdx2 is required for correct cell fate specification and differentiation of trophectoderm in the mouse blastocyst. Development.

[30] Wu G., Gentile L., Fuchikami T., Sutter J., Psathaki K., Esteves T.C., Arauzo-Bravo M.J., Ortmeier C., Verberk G., Abe K. (2010). Initiation of trophectoderm lineage specification in mouse embryos is independent of Cdx2. Development.

[31] Yagi R., Kohn M.J., Karavanova I., Kaneko K.J., Vullhorst D., DePamphilis M.L., Buonanno A. (2007). Transcription factor TEAD4 specifies the trophectoderm lineage at the beginning of mammalian development. Development.

[32] Grant J., Mahadevaiah S.K., Khil P., Sangrithi M.N., Royo H., Duckworth J., McCarrey J.R., VandeBerg J.L., Renfree M.B., Taylor W. (2012). Rsx is a metatherian RNA with Xist-like properties in X-chromosome inactivation. Nature.

[33] Lachmann A., Xu H., Krishnan J., Berger S.I., Mazloom A.R., Ma’ayan A. (2010). ChEA: Transcription factor regulation inferred from integrating genome-wide ChIP-X experiments. Bioinformatics.

[34] Duchene D.A., Bragg J.G., Duchene S., Neaves L.E., Potter S., Moritz C., Johnson R.N., Ho S.Y.W., Eldridge M.D.B. (2018). Analysis of phylogenomic tree space resolves relationships among marsupial families. Syst. Biol..

[35] Nilsson M.A., Arnason U., Spencer P.B., Janke A. (2004). Marsupial relationships and a timeline for marsupial radiation in South Gondwana. Gene.

[36] Chan J., Sharkey F.E., Kushwaha R.S., VandeBerg J.F., VandeBerg J.L. (2012). Steatohepatitis in laboratory opossums exhibiting a high lipemic response to dietary cholesterol and fat. Am. J. Physiol. Gastrointest. Liver Physiol..

[37] Hansen V.L., Faber L.S., Salehpoor A.A., Miller R.D. (2017). A pronounced uterine pro-inflammatory response at parturition is an ancient feature in mammals. Proc. Biol. Sci..

[38] Farrell G.C., van Rooyen D. (2012). Liver cholesterol: Is it playing possum in NASH?. Am. J. Physiol. Gastrointest. Liver Physiol..

[39] Yu L., Wei Y., Duan J., Schmitz D.A., Sakurai M., Wang L., Wang K., Zhao S., Hon G.C., Wu J. (2021). Blastocyst-like structures generated from human pluripotent stem cells. Nature.

[40] Dooley T.P., Mattern V.L., Moore C.M., Porter P.A., Robinson E.S., VandeBerg J.L. (1993). Cell lines derived from ultraviolet radiation-induced benign melanocytic nevi in Monodelphis domestica exhibit cytogenetic aneuploidy. Cancer Genet. Cytogenet..

[41] Robinson E.S., Dooley T.P., Williams K.L. (1998). UV-induced melanoma cell lines and their potential for proteome analysis: A review. J. Exp. Zool..

[42] Ma X., Dighe A., Maziarz J., Neumann E., Erkenbrack E., Hei Y.Y., Liu Y., Suhail Y., Kshitiz, Pak I. (2022). Evolution of higher mesenchymal CD44 expression in the human lineage: A gene linked to cancer malignancy. Evol. Med. Public Health..

[43] Anders S., Huber W. (2010). Differential expression analysis for sequence count data. Genome Biol..

[44] Chen E.Y., Tan C.M., Kou Y., Duan Q., Wang Z., Meirelles G.V., Clark N.R., Ma’ayan A. (2013). Enrichr: Interactive and collaborative HTML5 gene list enrichment analysis tool. BMC Bioinform..

[45] Ge S.X., Jung D., Yao R. (2020). ShinyGO: A graphical gene-set enrichment tool for animals and plants. Bioinformatics.

[46] Kuleshov M.V., Jones M.R., Rouillard A.D., Fernandez N.F., Duan Q., Wang Z., Koplev S., Jenkins S.L., Jagodnik K.M., Lachmann A. (2016). Enrichr: A comprehensive gene set enrichment analysis web server 2016 update. Nucleic Acids Res..

